# Practical Departments

**Published:** 1905-06-03

**Authors:** 


### PRACTICAL DEPARTMENTS.

### A SERVICEABLE DOORMAT.

The Lathina mat is a practical and excellent contrivance, and we do not hesitate to say that wherever it obtains a trial it will supersede the old-fashioned cocoanut fibre mat. We have put it to a lengthy test, and find that besides wearing well, its use has minimised the amount of dirt that is inevitably introduced into a public building in bad weather. The mat, as shown in our illustration, is formed of a framework of wood, across which are flat bars of the same material round which are closely wound, strands of cocoanut fibre. The mat has a firm and non-absorbent surface, against which the sides and soles of boots can be effectively cleaned. The dirt which lodges in the well in which the mat is laid, does not come in contact with it, as the framework is sufficiently raised to prevent contact with the floor. A stiff brush easily removes the small amount of mud which adheres to the surface. We would recommend that the floor of the well should be lined with linoleum. This will improve the appearance, and facilitate the speedy and easy removal of dirt, and thus enhance the hygienic value of the contrivance. The Lathina mat has the further recommendation of cheapness. It can be obtained from the Lathina Mat Company, Fleet, Hants.

